# Host-Microbe Interactions in Microgravity: Assessment and Implications

**DOI:** 10.3390/life4020250

**Published:** 2014-05-26

**Authors:** Jamie S. Foster, Raymond M. Wheeler, Regine Pamphile

**Affiliations:** 1 Space Life Science Lab, University of Florida, 505 Odyssey Way, Merritt Island, FL 32953, USA; E-Mail: reginep116@ufl.edu; 2 Kennedy Space Center, FL 32899, USA; E-Mail: raymond.m.wheeler@nasa.gov

**Keywords:** host-microbe interactions, microgravity, microbiome, space flight

## Abstract

Spaceflight imposes several unique stresses on biological life that together can have a profound impact on the homeostasis between eukaryotes and their associated microbes. One such stressor, microgravity, has been shown to alter host-microbe interactions at the genetic and physiological levels. Recent sequencing of the microbiomes associated with plants and animals have shown that these interactions are essential for maintaining host health through the regulation of several metabolic and immune responses. Disruptions to various environmental parameters or community characteristics may impact the resiliency of the microbiome, thus potentially driving host-microbe associations towards disease. In this review, we discuss our current understanding of host-microbe interactions in microgravity and assess the impact of this unique environmental stress on the normal physiological and genetic responses of both pathogenic and mutualistic associations. As humans move beyond our biosphere and undergo longer duration space flights, it will be essential to more fully understand microbial fitness in microgravity conditions in order to maintain a healthy homeostasis between humans, plants and their respective microbiomes.

## 1. Introduction

Spaceflight impacts all living organisms from their genome to physiome. Whether it is the reduction in gravity, increased exposure to low dose radiation, chronic elevated CO_2_ levels or time dislocation, the physical factors associated with the spaceflight environment presents a unique set of stresses on biological systems. Of these physical factors, the reduction in gravity, or microgravity, is one of the most widely studied [1,2,3,4,5,6,7]. Gravity represents one of the most constant evolutionary drivers of life on Earth [8,9], and understanding how organisms respond to the absence of this ever-present force not only provides information on how gravity has shaped life on Earth, but also provides key insight into potential mechanisms to mitigate the negative effects of microgravity during spaceflight. The large-scale physiological effects of microgravity-induced stress on animals and plants are relatively well known. For example, in humans, microgravity conditions result in bone loss, upwards to 3% per month [4,10,11], permutations to both the adaptive and innate immune systems [12], and an increased potential risk of bacterial and viral infections [13,14]. Plants also have an increased susceptibility to pathogenic colonization in microgravity [15], as well as altered rates of cell division, changes in morphological structures of cell tissues, such as root hairs, and induced chromosomal aberrations [16,17,18]. Although these overarching phenotypes associated with microgravity exposure have been observed for decades, ascertaining whether the phenotypes are specific to microgravity or other environmental variables, as well as the underlying etiology of these effects at the cellular and biomolecular level are not yet fully delineated.

**Figure 1:**
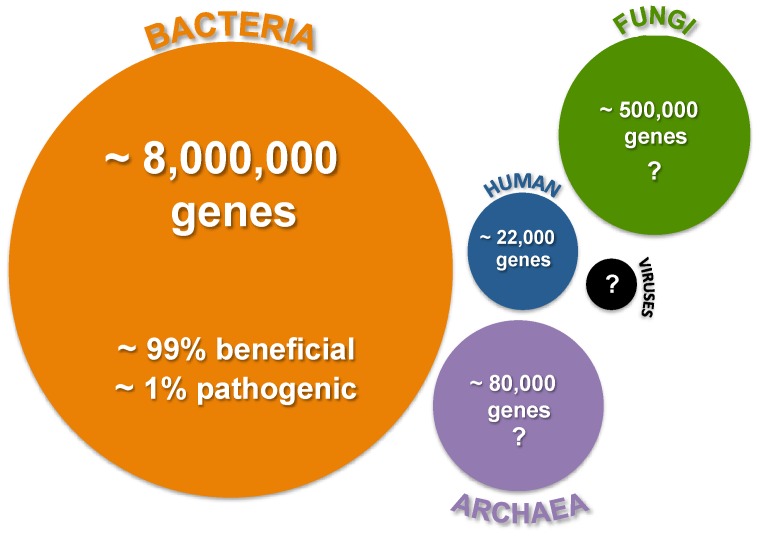
Overview of the complexity of human microbiome. Circle size reflects the approximate relative abundance of the various microbes known to associate with humans. Relative gene abundance is derived from the human microbiome sequencing project [19,22]. Question marks reflect uncertainty or potential underestimation of gene abundance.

Compounding our understanding of the mechanisms underlying these physiological effects is the relatively unknown impact of microgravity on the microbiome associated with eukaryotic organisms. A microbiome is typically defined as the sum of the microbes, genomes and community interactions that occur in a particular environment. The term was first applied to humans to understand the ecology of those microbes that interact with the body [19] but has been quickly adopted to represent a holistic approach to understanding the connectivity and interactions within any complex host-microbe association [20]. In humans, the recent human microbiome sequencing project has revealed that the collective metagenome of those microbes that associate with humans dwarfs the size of the human genome and contains a consortia of bacteria, archaea, fungi and viruses, the specific contributions of which are not yet fully delineated (Figure 1) [21]. Initial surveys indicate that for every one human gene there are approximately 360 bacterial genes, thereby providing humans with millions of genes of additional metabolisms and cellular activities [22,23].

Over the past decade, there has been a paradigm shift in our understanding of the importance the microbiome plays in maintaining plant and animal health [22,24]. Aspects previously attributed to the host are now recognized as being the result of interactions with microbes [22,25,26]. For example, analyses of germ-free and normally colonized mice have shown that the gut microbiota is responsible for most of the metabolites found in mammalian blood [27]. Additionally, some species of bacteria within human gut have been recently correlated to the onset of several autoimmune diseases, such as rheumatoid arthritis and Type 1 diabetes [28,29,30,31]; colorectal cancers [32]; and obesity [33] suggesting that perturbations to the gut environment (e.g., antibiotic treatments) can increase the potential risk of disease. However, in many of these same cases, modulations of the gut microbiota and probiotic therapies have been shown to attenuate the condition and treat the host organism [24].

Together, these studies indicate that stable diverse microbial communities and functional gene redundancies can provide resiliency to the host’s microbiota to withstand temporary community disturbances and potentially resist invasion by exogenous pathogenic microbes [24,34]. However, continuous or regular perturbations to the environment, such as exposure to microgravity, may result in loss of biodiversity or extirpation (*i.e.*, the extinction of a species in localized areas of the microbiome), potentially driving the community towards disease.

Although the short- and long-term resilience (*i.e.*, susceptibility of community to disturbances or extirpation) of animal and plant microbiomes in response to microgravity is virtually unknown, microbial exchange and transfers do occur between microbiomes during spaceflight [35,36] and there is increasing evidence that microgravity induces physiological and developmental changes within specific host-microbe interactions as discussed below. Assessing the impact of microgravity on a host organism and its microbiota requires a comprehensive analysis that includes the study of both pathogenic and mutualistic bacteria as cultures and *in situ* to fully understand the requirements needed to maintain host health in the space flight environment.

## 2. Spaceflight and Simulated Microgravity Environments

Experimentally testing the effects of microgravity on host-microbe interactions at the cellular and biomolecular level often requires a two-pronged approach. First, in ideal situations the impact of microgravity on both host and symbiont physiology can be investigated under a high-quality microgravity (10^−6^ g) environment, such the International Space Station U.S. National Laboratory [5,37]. However, logistics with regard to access to the station, astronaut time, the need for specialized equipment and overall costs can impose limitations on experimental design and reproducibility. A second approach to microgravity research has been the development of Earth-based systems to simulate or model the microgravity environment. Examples include drop towers at various NASA facilities, and parabola flights in aircraft (e.g., Zero-G), which can provide several seconds of microgravity, albeit at a lower quality (10^−3^ g). A new frontier in microgravity research is the emergence of numerous commercial suborbital companies such as Virgin Galactic, XCOR Aerospace, Blue Origin, and Masten Space Systems that in the next few years will offer lower flight cost, and more frequent and rapid flights rates for microgravity research.

Although these alternative approaches may provide up to several minutes of microgravity exposure, other ground-based tools have emerged over the past few decades that can model the low-shear, low-turbulent effects of microgravity over longer periods of time and will be discussed in this review. Two of these technologies, the clinostat and rotating wall vessel (RWV), provide a relatively low cost approach to investigate the cumulative effects of microgravity on both pathogenic and mutualistic microbes [1,4,13,38,39,40,41]. Both technologies facilitate constant rotation that is perpendicular to the gravitational vector thus maintaining the cell cultures in a low shear suspension where the hydrodynamic forces offset gravitational settling, thus enabling the organism to be in a simulated microgravity state [1,39].

Rotating bioreactors can effectively replicate the low shear environments of many parts of the human body where bacteria typically associate, such as the brush-border of epithelial cells, one of the most common sites for host-microbe interactions in animals [42,43]. Although initially the reactors were used for bacterial suspensions, the technology has been successfully adapted to examine more complex aspects of host-microbe interactions. For example, the RWV bioreactors have been used to stimulate the formation of three-dimensional eukaryotic cell cultures that more realistically approximate the function and cellular tissue structure an exogenous microbe might encounter. More recently, RWV bioreactors have been used to look at the impact of microgravity on the colonization of host tissues *in situ*, where both the host and symbiotic partners are co-incubated within the rotating vessels [41,44,45]. Although the shear forces increase when working with larger organisms in the RWV bioreactors, previous modeling experiments have shown that the fluid shear levels of objects up to 3 mm mimic those typically seen by microbes when they associate with host tissues [46,47].

## 3. Impact of Microgravity on Animal-Microbe Associations

### 3.1. Pathogenic Interactions with Animals

Over the past few decades, numerous studies have examined the effects of both natural and modeled microgravity on pathogenic microbes revealing several pronounced physiological responses [1,7,37,40,44,48,49]. These microgravity-induced changes include: changes in growth rate and higher cell densities; increased membrane integrity [7,45,50,51]; differential secondary metabolite production [52]; elevated transfer rates of genetic material between cells [53,54] and increased biofilm formation [5,6,55,56].

In addition to these basic physiological responses, several well-characterized pathogenic microbes have also exhibited changes at the molecular level by altering expression of genes known to be associated with virulence in microgravity including: *Salmonella enterica* serovar Typhimurium cultures; *Escherichia coli*; and *Pseudomonas aeruginosa* [5,57,58,59,60]. In *S. typhimurium* cultures grown in both space flight and ground-based analog microgravity environments, there was faster colonization of mouse spleen and liver tissues and shortened time-to-death rates [5,57]. This enhanced pathogenicity in *S. typhimurium* has also been correlated to the overall fitness of the cultures in microgravity, including increased resistance to antibiotics, pH, temperature and osmotic stresses [1,3,37,55]. Transcriptional and proteomic analysis of microgravity-exposed *S. typhimurium* has revealed the differential expression of numerous genes and proteins associated with stress responses and virulence, such as lipopolysaccharide synthesis enzymes, cell invasion genes, and the global regulator Hfq [5,61,62]. Hfq, a RNA binding protein known to have a diverse role in bacterial physiology [63], has been shown to be essential for *S. typhimurium* virulence [64] as well as numerous other pathogenic microbes [59,62,65,66]. Of the many genes associated with microgravity exposure, the global Hfq regulator was one of the few differentially expressed genes to be identified in other pathogenic species, such as *Pseudomonas aeruginosa* and *Staphylococcus aureus* [5,59,62], suggesting it plays a diverse and critical role in mediating the microgravity response in bacteria.

Although *S. typhimurium* clearly exhibited increased virulence in both natural and modeled microgravity, the response was environment-dependent [49]. Altering the inorganic salt concentration of the surrounding media effectively inhibited the enhanced virulence [49] suggesting that it may be possible to mitigate aspects of pathogenicity during space flight. Additionally, recent examination of the ability of several clinical pathogens to infect an animal host *in situ*, where both organisms were maintained under both space flight and modeled microgravity conditions, showed an overall decrease in virulence [44]. In this study, four diverse pathogens including: *Listeria monocytogens*, a common food-borne pathogen; *Staphylococcus aureus*, associated with skin and respiratory infections; *Enterococcus faecalis*, a prevalent antibiotic resistant species associated with gastrointestinal track; and *Candida albicans,* a fungus known to associate with skin and mucus membranes, were incubated with the nematode *Caenorhabditis elegans* for up to 48 h and then the host was assessed for viability. The overall decrease in virulence may be the result of basic morphological (*i.e.*, coccoid *versus* filamentous) or motility differences between the microbial ecotypes that alter the low-shear environment of space flight, thus impacting the ability of the pathogen to colonize and infect host tissues [67]. These results may also simply reflect host-specific or even tissue-specific differential gene expression in response to various pathogens under microgravity conditions. Such studies reinforce the need to fully assess the environmental or community characteristics that predispose or drive host-microbe interactions towards disease.

### 3.2. Mutualistic Interactions with Animals

**Figure 2:**
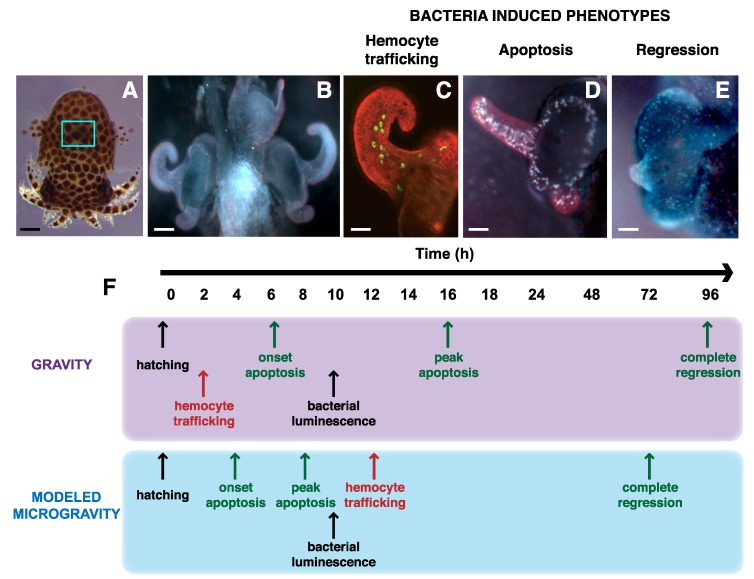
Impact of microgravity on the developmental time line of mutualistic symbiosis between the squid *Euprymna scolopes* and bacterial partner *Vibrio fischeri*. (**A**) Juvenile squid just after hatching. Blue rectangle marks the location within the host mantle cavity of light organ, the site of symbiosis. Bar, 0.25 mm (**B**) Light organ at hatching showing the elongated surface epithelium that forms appendage like structures on either side of the light organ. Bar, 75 μm. (**C**) One half of light organ depicting the movement of hemocytes (green) moving into the blood sinus contained within the surface epithelium upon exposure to bacteria. Bar, 30 μm. (**D**) Light organ exposed to bacterial lipopolysaccharide showing pronounced cell death staining pattern along the superficial epithelium. Bar, 30 µm. (**E**) Image of one half of light organ depicting the loss of the superficial epithelial appendage structures 96 h after colonization with *V. fischeri*. Bar, 30 µm. (**F**) Exposure to microgravity alters the developmental time line of the symbiosis under normal gravity and modeled microgravity conditions using a RWV bioreactor. Events listed in black do not change under microgravity conditions. Those events in red are delayed during modeled microgravity, where as those events in green are accelerated.

Although disease-causing microbes represent a potential risk to astronauts in microgravity conditions, it is also essential to have a corresponding understanding of mutualistic microbes to learn what drives microbial fitness in the spaceflight environment and how to maintain a healthy homeostasis between humans and their microbiome. To address these issues, one model system has recently emerged to assess the interactions between animal cells and beneficial microbes *in situ* under microgravity conditions (Figure 2). The binary symbiosis between the Hawaiian bobtail squid *Euprymna scolopes* and its luminescent partner *Vibrio fischeri* was established over two decades ago as a tractable model for examining beneficial animal-microbe interactions [68,69].

The site of symbiosis occurs in the host light organ, located within the squid’s mantle cavity (Figure 2A). The light organ contains a series of superficial invaginations that form long ciliated ducts, which terminate into a series of epithelial lined crypt spaces where the bacteria eventually reside and grow. On the surface of the light organ is a superficial field of epithelial cells overlaying a blood sinus that form two appendage-like structures and are critical for facilitating the initiation of the symbiosis (Figure 2B). Studies have revealed that the colonization and maintenance of the light organ symbiosis is highly specific and requires a complex exchange of signaling molecules and cell–cell communication that is in part mediated by the host immune system [70,71].

One of the initial signals involved in establishing the symbiosis is the use of micro-associated molecular pattern (MAMP) molecules, such as lipopolysaccharides (LPS) and peptidoglycan. Originally characterized in pathogenic associations, LPS and peptidoglycan have been recently shown to be critical for maintaining gut homeostasis in mammals and insects [25,72]. The squid–vibrio symbiosis is initiated when upon hatching from their egg case the squid is exposed to bacteria-rich seawater. Peptidoglycan that is shed from these surrounding microbes induces mucus to be secreted by the surface epithelial appendage cells of the host light organ. The accumulation of mucus selectively enriches *V. fischeri* from the other surrounding microbes and within 2–3 h after hatching the *V. fischeri* begins to colonize the light organ by entering pores on the surface of the light organ then migrating through ducts until they reach the crypt spaces. During the colonization of the light organ, the bacteria induce a series of developmental events in the host squid that eventually results in a remodeling of the host light organ structure.

One of the first symbiont-induced phenotypes in the host squid is the activation of the host’s innate immune response [69,71,73]. With two hours of exposure to *V. fischeri* cells, host-derived hemocytes, a macrophage-like cell, are trafficked into the blood sinus of the superficial epithelial cell appendage (Figure 2C). The precise role these host immune cells play in the symbiosis is not well defined, however, it is thought they facilitate the regression of superficial epithelial cells once colonization has been initiated [69]. The regression event is triggered by a bacteria-induced apoptosis event by the synergistic activity of the bacterial MAMP molecules LPS and a derivative of peptidoglycan, tracheal cytotoxin (Figure 2D) [74,75]. The apoptosis event peaks 16 h after exposure to the *V. fischeri* MAMPS and regression of the epithelial appendages is visible by 24 h and takes approximately 96 h for complete regression (Figure 2E) [76,77,78].

In modeled microgravity conditions, where both partners are co-incubated using RWV bioreactors, the bacteria-induced developmental time line in the host light organ is altered (Figure 2F). One of the first observed differences is that the trafficking of host-derived hemocytes into the light organ blood sinus, which normally occurs within two hours of bacteria exposure, is delayed until 12 h in modeled microgravity [41]. The cause for the delay is not known, but the amount of hemocyte cells in the blood sinus is significantly lower than in gravity controls suggesting that the low-shear, modeled microgravity environment suppresses the activation and transport of hemocytes to the light organ [41]. One potential explanation is that the squid hemocytes proliferation or cytokine signaling may be reduced in microgravity-like conditions. Similar results have been seen in mammalian immune systems during space flight [79,80].

Another key phenotype that changes in the squid–vibrio system during modeled microgravity is the acceleration of the light organ morphogenesis; specifically, the LPS-induced apoptotic cell death event throughout the superficial field of epithelial cells. Normally, the LPS triggered apoptosis event peaks approximately 16 h after bacterial exposure, however, in modeled microgravity it occurs around 8 h after exposure [41]. The epithelial cells comprising this structure appear to have an increased sensitivity to extracellular LPS in modeled microgravity conditions, although the mechanism is not fully understood [45]. The acceleration of the LPS-induced apoptosis event may be tied to the global regulator Hfq, which similar to *S. typhimurium* and *P. aeruginosa* is down regulated in *V. fischeri* during modeled microgravity conditions [45]. Additionally, mutants defective in the *hfq* gene exhibited attenuated levels of apoptotic cell death in microgravity although the timing was still accelerated compared to gravity controls [45]. Experiments are currently underway to examine changes in microgravity-induced gene expression of wild-type *V. fischeri* and mutants defective in the *hfq* gene to assess the impact of modeled microgravity on the symbiont transcriptome (J. Foster unpublished).

## 4. Impact of Microgravity on Plant-Microbe Associations

As humans move beyond Earth’s biosphere and undergo the long-term habitation of space, life support systems will inevitably require plants. Understanding the physical and genetic impact of microgravity on plant physiology and their associated microbes will be critical [16,18]. Much like the animal microbiome, plants are also colonized by trillions of microbial cells that form complex and metabolically diverse ecosystems influencing their health and growth [81,82]. In terrestrial systems, these beneficial microbes are typically associated with the rhizosphere, a narrow zone surrounding the plant roots. Plants can exude up to 21% of their photosynthetically fixed carbon into the root–soil interface [83], which can influence the microbial activity and diversity, thereby enhancing seed germination rates, nutrient uptake, plant growth and development [82,84]. Recent studies have shown that plants actively recruit disease-suppressive microbes that can protect the host from various infections through the production specific molecules, such as nonribosomal peptide synthases [85,86], and boost the plant immune system [87]. For example, in *Arabidopsis thaliana*, the rhizobacterium *Pseudomonas fluorescence* can induce the induced systemic resistance (ISR) response of the host by the apoplastic secretion of low molecular weight molecules that locally suppress flagellin-triggered immune responses [88]. Activation of ISR can accelerate defense-associated gene expression in the host, thus priming the plant immune system and facilitating resistance to a wide range of pathogens [89].

In spaceflight, however, the structural complexity and species richness of the plant rhizosphere can be reduced due to the limitations on using friable soils in microgravity and the lack of natural water drainage, which can result in poor aeration in the root zone [90,91]. Plants cultivated in space flight conditions have been grown in various media, including agar plates, growth pouches, absorbent “floral foams”, and in sub-irrigated porous arcillite (calcined clay chips) [92,93]. These studies have often used specialized growth chambers (e.g., Astroculture, the Biomass Production System, Lada, and others), which typically control light, humidity, carbon dioxide and regulated temperature [18,90,92,94,95,96,97,98]. Some of these chambers, such as the Russian Svet (on Mir) and the Lada on the International Space Station (ISS) are open to the cabin air [97] and hence exposed to the organic volatiles and very high CO_2_ levels of the cabin air, which can affect the plants in ways unrelated to microgravity [99,100]. It is not unreasonable to assume that these other space environment effects could then also influence host-microbe interactions.

Several investigations have revealed that plants grown in microgravity exhibit an increased susceptibility to plant pathogens [15,96,101,102]. For example, soybeans cultivated under space flight conditions in the presence of the pathogenic fungus *Phytophthora sojae* showed high levels of disease symptoms (*i.e.*, browning and maceration of the roots) compared to ground controls. Additionally, increased levels of fungal structures penetrating the vascular tissue of the plant were also observed suggesting that under microgravity conditions the pathogen more effectively colonized the host tissues [96]. Microgravity exposure has also been shown to negatively impact plant cell wall integrity, as both cell wall regeneration and lignin biosynthesis can be repressed under spaceflight conditions thereby facilitating colonization of fungal pathogens [18,102]. This increase in pathogenesis during spaceflight may reflect the disruption to the normal healthy rhizosphere microbiome.

Although the effects of microgravity appear to increase susceptibility of the plant host to disease, less is known of its impact on associations with beneficial microbes. One of the few known examples includes the symbioses between nitrogen-fixing bacteria and leguminous plants. Nitrogen fixation is a critical bacterial metabolism for future life support systems to help cycle inert dinitrogen to the more usable ammonia [103], however, the effects of microgravity on the major stages of root nodule formation and colonization are not well known. Early studies have shown that under modeled microgravity conditions, *Rhizobium leguminosarum* cells can increase binding of succinate, a key molecule in the differentiation of the bacteria to bacteroids, a distinct cell-type capable of fixing nitrogen [103,104]. This increased binding, however, did not alter the developmental time line of the bacteroid formation and there were no statistical differences between microgravity and gravity controls [103]. Similar experiments have been since replicated during spaceflight for longer durations using the partners *Medicago truncatula*, the barrel clover, and the bacterial symbiont *Sinorhizobium meliloti* (G. Stutte and M. Roberts unpublished). No developmental delays were observed between spaceflight and 1 × g gravity controls conditions. The *S. meliloti* cells were able to effectively colonize and induce normal nodule formation in the plant host suggesting this symbiosis may play an important role in future life support systems (G. Stutte and M. Roberts unpublished). Although additional work is required to characterize the impact of spaceflight on these rhizobia-legume symbioses and other members of the plant microbiome, especially at the genome-level, these initial results suggest that microgravity does not impair the initial colonization and cell–cell communication required by the host and microbial association. As the plant microbiome can, in turn, directly influence the human microbiome through the ingestion of raw plants or through environmental interactions in a closed life support system, it will become critical to expand our understanding of the effects of microgravity on these key plant–microbe interactions.

## 5. Summary

The recent advance in our understanding of human and plant biology with respect to their microbiomes represents a new frontier for space biology research [105]. It is increasingly clear that astronauts, and other eukaryotic hosts, should be considered as metaorganisms, whose crosstalk and interactions with their microbes at the genetic and cellular level have redefined our definition of health and disease [106]. As manned exploration of space continues beyond the biosphere, it will be critical to examine the stability and resiliency of the human and plant metaorganism to spaceflight-induced stresses, including microgravity. For example, if keystone microbes are lost in the host community, this may result in a shift in species diversity and richness of the microbiome, thus potentially facilitating the rise of opportunistic pathogens that might otherwise be contained by the normal microbial community.

To the microbe, eukaryotes simply represent a complex environment in which it must perpetually navigate and adapt to maintain its fitness [22]. This adaptation can occur through horizontal gene transfer events, and recent sequencing evidence suggests that in humans, the microbiome is a hotspot for genetic transfer, facilitating the exchange of antibiotic resistance, food digestion and nutrient metabolism genes that may provide the microbe a competitive advantage within the host community [3,107]. Understanding the nature and frequency of such events, as well as the overall stability of the microbiome under microgravity conditions, will not only help elucidate how microgravity alters this dynamic microbial landscape but will help enable the formation of new strategies for maintenance and potential restoration of the healthy microbiome during spaceflight.
